# A *Ma10* gene encoding P‐type ATPase is involved in fruit organic acid accumulation in apple

**DOI:** 10.1111/pbi.13007

**Published:** 2018-11-01

**Authors:** Baiquan Ma, Liao Liao, Ting Fang, Qian Peng, Collins Ogutu, Hui Zhou, Fengwang Ma, Yuepeng Han

**Affiliations:** ^1^ Key Laboratory of Plant Germplasm Enhancement and Specialty Agriculture Wuhan Botanical Garden of the Chinese Academy of Sciences Wuhan China; ^2^ State Key Laboratory of Crop Stress Biology for Arid Areas/Shaanxi Key Laboratory of Apple College of Horticulture Northwest A&F University Yangling Shaanxi China; ^3^ Sino‐African Joint Research Center Chinese Academy of Sciences Wuhan China; ^4^ Graduate University of Chinese Academy of Sciences Beijing China

**Keywords:** *Malus domestica*, fruit acidity, P‐type ATPase, transcriptome analysis, candidate gene association mapping

## Abstract

Acidity is one of the main determinants of fruit organoleptic quality. Here, comparative transcriptome analysis was conducted between two cultivars that showed a significant difference in fruit acidity, but contained homozygous non‐functional alleles at the major gene *Ma1* locus controlling apple fruit acidity. A candidate gene for fruit acidity, designated *M10*, was identified. The *M10* gene encodes a P‐type proton pump, P_3A_‐ATPase, which facilitates malate uptake into the vacuole. The *Ma10* gene is significantly associated with fruit malate content, accounting for ~7.5% of the observed phenotypic variation in apple germplasm. Subcellular localization assay showed that the Ma10 is targeted to the tonoplast. Overexpression of the *Ma10* gene can complement the defect in proton transport of the mutant YAK2 yeast strain and enhance the accumulation of malic acid in apple callus. Moreover, its ectopic expression in tomato induces a decrease in fruit pH. These results suggest that the *Ma10* gene has the capacity for proton pumping and plays an important role in fruit vacuolar acidification in apple. Our study provides useful knowledge towards comprehensive understanding of the complex mechanism regulating apple fruit acidity.

## Introduction

Sour taste or acidity is an important component of the organoleptic quality of fruits. Fruit acidity is attributed to the accumulation of organic acids whose acidity is commonly associated with their carboxyl group. In general, the predominant organic acids in most fruits are citric and malic acids, with unequal acidic taste. Malic acid has a more prolonged sour sensation in the mouth and thus displays a higher level of sourness compared with citric acid (Ramos Da Conceicao Neta *et al*., 2007). Hence, fruit sourness level depends on the content and type of organic acids.

In fruit cell, malic acid is mainly synthesized in the cytosol by carboxylation of phosphoenolpyruvate (PEP) through the catalysation of phosphoenolpyruvate carboxylase (PEPC) and malate dehydrogenase (MDH), and it can also be degraded via decarboxylation catalysed by MDH and NADP‐malic enzyme (NADP‐ME) (Sweetman *et al*., 2009). Citric acid is produced by the tricarboxylic acid (TCA) cycle in the mitochondria, and mitochondrial citrate synthase (mCS) is the crucial enzyme controlling citrate synthesis. Citrate can be degraded through the activities of two enzymes, aconitase (ACO) and NAD‐dependant isocitrate dehydrogenase (NAD‐IDH), in either the mitochondria through the TCA cycle or the cytosol through the GABA shunt that leads to succinate synthesis (Katz *et al*., 2011; Sadka *et al*., 2001). Since organic acids in fruit cells are mainly stored in the vacuole, vacuolar storage is supposed to be critical for the accumulation of organic acids in fruit cells. For example, malate accumulation is mainly driven by vacuolar storage (Etienne *et al*., 2013). In contrast, citrate accumulation is mainly controlled by metabolism because uptake of citrate into the vacuole may be limited due to the low activity of its transport system in the vacuolar membrane (Hafke *et al*., 2003).

Vacuolar storage of organic acids requires many transporters and proton pumps in the vacuolar membrane, such as tonoplast dicarboxylate transporter (tDT) (Emmerlich *et al*., 2003), aluminium‐activated malate transporter (ALMT) (Meyer *et al*., 2011), the vacuolar‐type H^+^‐ATPase (V‐ATPase) (Aprile *et al*., 2011) and the vacuolar H^+^ pumping pyrophosphatase (V‐PPase) (Suzuki *et al*., 2000). More recently, two additional classes of genes encoding tonoplast transport proteins have also been identified to regulate the accumulation of organic acids in fruit. One is *CmPH* encoding the PIN H^+^/auxin transporter and a four amino‐acid duplication in the coding region causing the acidity difference between acidic and dessert melons (Cohen *et al*., 2014). Another is a class of two P‐ATPase proton pumps, PH5 and PH1, which directly interact with each other and form a heteromeric complex to promote vacuolar acidification in petunia flower (Faraco *et al*., 2014; Verweij *et al*., 2008). Later, a *PH5*‐like H^+^‐ATPase gene has been found to show an extremely low expression in acid‐free fruit of orange, thus, it is presumably involved in the regulation of vacuolar storage of citrate in fruit (Shi *et al*., 2015). Besides participating in vacuolar acidification, the tonoplast P‐ATPase TT13 (AtAHA10) can also generate the driving force for the transport of proanthocyanidin precursors to the vacuole in *Arabidopsis*, (Appelhagen *et al*., 2015). In addition, increasing evidence shows that transcription factor genes participate in the regulation of the proton pump gene expression. For example, a petunia WRKY gene *PH3* and an apple *MYB1* gene regulate the transcription of P‐ATPase (Verweij *et al*., 2016) and V‐ATPase (Hu *et al*., 2016) respectively. An ethylene response factor (ERF) has been shown to interact with V‐ATPase to regulate citrate accumulation in citrus fruit (Li *et al*., 2016a). Overall, the mechanism controlling organic acid metabolism and accumulation in fruits is complex and still remains unclear.

Apple (*Malus *× *domestica* Borkh.) is one of the most important fruit trees in temperate regions, and its draft genome sequence has been released (Velasco *et al*., 2010). In apple fruit, the predominant organic acid is malate that accounts for approximately 90% of the total organic acid content (Ma *et al*., 2015a). In the past two decades, several quantitative trait loci (QTLs) for apple fruit acidity were identified on linkage groups (LGs) 8, 10, 13, 15, 16, and 17, with one (designated *Ma*) on the top of LG16 having a major effect (Kenis *et al*., 2008; Liebhard *et al*., 2003; Maliepaard *et al*., 1998; Zhang *et al*., 2012). Recently, the major QTL in the *Ma* locus was found to encode aluminium‐activated malate transporter (Bai *et al*., 2012; Khan *et al*., 2013), and fruit acidity is hypothetically determined by a gene network in which the *Ma1* gene is a key mediator (Bai *et al*., 2015). However, several apple cultivars containing two non‐functional mutant alleles of the *Ma1* locus also have lower pH values in fruit, which suggests that the *Ma1* gene is not the only genetic determinant of fruit acidity in apple (Ma *et al*., 2015b). Moreover, in addition to the *Ma1* gene, another major QTL for fruit acidity was identified on LG8 in different segregating populations (Kenis *et al*., 2008; Ma *et al*., 2016; Sun *et al*., 2015). Hence, our knowledge on genetic basis of the biosynthesis and accumulation of malic acid in apple fruit is still limited.

Transcriptome analysis is becoming an effective and fast method to identify genes controlling the important agronomic traits in fruit crops (Lee *et al*., 2012; Liu *et al*., 2009). We recently investigated the genetic diversity for organic acid content in apple germplasm and found that a cultivar Belle de Boskoop accumulates high level of malate in fruit although it contains two homozygous mutant alleles in the *Ma1* locus, *ma1ma1* (Ma *et al*., 2015b). In this study, analysis of fruit transcriptome profiles of ‘Belle de Boskoop’ was conducted to identify gene(s) other than *Ma1* that are responsible for apple fruit acidity. A gene encoding the P‐ATPase proton pump, designated *Ma10*, was identified to control fruit acidity. This study will facilitate our understanding of the complex gene network regulating fruit acidity in apple.

## Results

### Transcriptome sequencing, assembly and functional annotation

*Ma1* is a major gene controlling apple fruit acidity, and a single nucleotide substitution of G with A in the last exon causes a premature stop codon, resulting in a loss‐of‐function allele *ma1* (Bai *et al*., 2012; Khan *et al*., 2013). Our previous study revealed that two cultivars, ‘Aifeng’ (AF) and ‘Belle de Boskoop’ (BSKP), contain homozygous mutant alleles, *ma1ma1*, but they show a significant difference in fruit malate accumulation, bearing non‐acidic and acidic fruits respectively (Ma *et al*., 2015b). To identify genes other than *Ma1* for fruit acidity, fruit transcriptome was compared between ‘AF’ and ‘BSKP’. Four RNA‐seq libraries were constructed and sequenced for fruit samples of ‘AF’ and ‘BSKP’ at two developmental stages, 30 and 90 DAFB. For ease of description, RNA‐seq libraries for fruit samples of ‘AF’ at 30 and 90 DAFB were designated AF1 and AF2 respectively, while BSKP1 and BSKP2 representing RNA‐seq libraries for fruit samples of ‘BSKP’ at 30 and 90 DAFB respectively (Table 1).

**Table 1:** Summary of RNA‐seq data for fruit samples of two cultivars, Aifeng (AF) and Belle de Boskoop (BSKP) at two developmental stages

Library	Variety	Stage (DAFB)	No. of raw reads	No. of clean reads	No. of clean reads mapped	Size of clean reads (Gb)	Q30 (%)	GC content (%)
AF1	AF	30	63 713 444	57 393 018	47 197 418	5.74	90.28	47.05
AF2		90	57 518 056	52 344 236	43 165 324	5.24	90.59	46.28
BSKP1	BSKP	30	66 152 318	59 958 220	48 444 402	6.00	90.54	46.55
BSKP2		90	64 766 890	58 685 724	47 806 215	5.86	90.48	46.57

The Q30 value ranged from 90.28% to 90.59%, suggesting the high quality of RNA‐seq data. The GC content of RNA‐seq reads ranged from 46.28% to 47.05%. The number of raw reads for each RNA‐seq library ranged from 57 518 056 to 66 152 318, with an average of 63 037 677. After removing adapter, empty reads and low quality sequences, the number of clean reads per library ranged from 52 344 236 to 59 958 220, and approximately 81.74% of the clean reads were mapped to the apple genome (Velasco *et al*., 2010). Reads mapped to unique and multiple sites of the reference genome were designated uni‐ or multi‐reads respectively. For each library, approximately 80.7% and 19.3% of the clean reads were identified to be uni‐reads and multi‐reads respectively.

**Figure 1:**
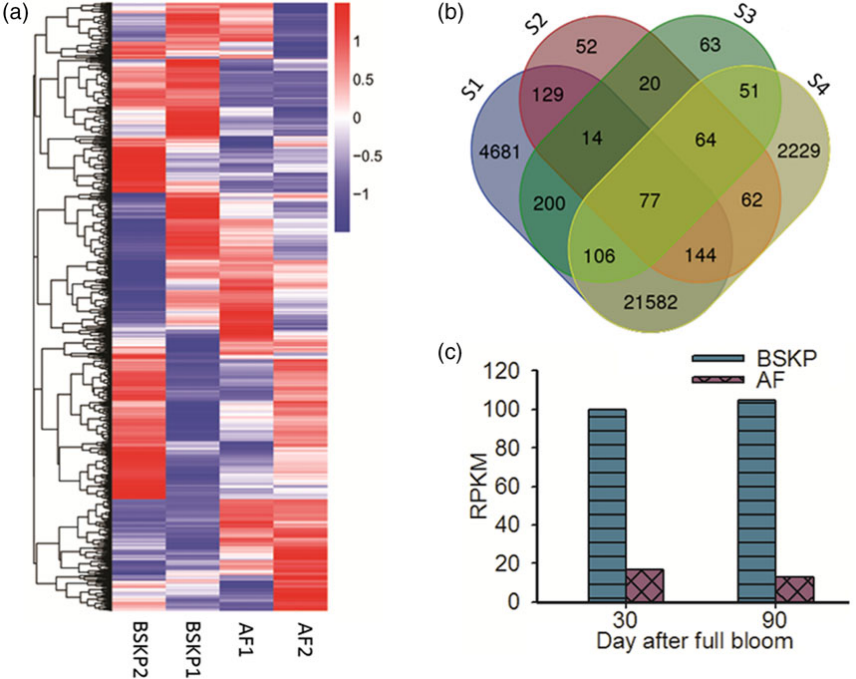
Transcriptome analysis of fruit samples of two apple cultivars at two developmental stages. (a) Hierarchical clustering of transcript accumulation between the four fruit samples. The suffixes of 1 or 2 added to the end of abbreviation of cultivar names represent 30 or 90 days after full bloom respectively. (b) Venn diagram showing identification of the candidate genes for malic acid content in apple. S1 and S4 represent genes that show no significant difference in expression level between fruits of ‘AF’ or ‘BSKP’ at 30 and 90 DAFB respectively. S2 and S3 indicate genes that show down‐regulation in fruits of ‘AF’ at 30 and 90 days after full bloom respectively, compared with ‘BAKP’. (c) Digital gene expression level of the candidate gene *Ma10* for fruit acidity in ‘BSKP’ and ‘AF’.

A total of 31 905 genes were identified in all fruit samples tested. Among these genes, 27 931, 29 391, 29 006 and 26 806 genes were expressed in AF1, AF2, BSKP1 and BSKP2, respectively (Figure 1a). Of the 31 905 genes expressed in fruit, 3249 displayed twofold or greater difference in expression level between any two fruit samples. Cluster analysis of the expression pattern revealed that the 3249 differentially expressed genes could be divided into 26 subgroups, each of which displayed a unique gene expression profile in fruit samples tested (Figure S1).

### Identification of candidate gene(s) for apple fruit acidity

In this study, we focused on the identification of candidate genes that were highly expressed in acidic fruit, but lowly expressed in non‐acidic fruit. Digital gene expression (DGE) analysis revealed that 562 and 592 genes showed significant down‐regulation in fruits of ‘AF’ at 30 and 90 DAFB respectively, compared with fruits of ‘BSKP’ at the same stage (Figure 1b). Of these differentially expressed genes, 175 were down‐regulated in fruits of ‘AF’ at both 30 and 90 DAFB compared with ‘BSKP’, thus, designated common down‐regulated genes. In addition, our previous study showed that ‘BSKP’ had high levels of malic acid accumulation in fruit throughout the developmental stages, whilst a low level was observed for ‘AF’ (Ma *et al*., 2015b). This suggests that the candidate genes for fruit acidity were likely to have a consistent expression in fruits of ‘BSKP’ and ‘AF’ during the developmental stages. DGE analysis revealed that 26 933 and 24 315 genes showed no significant difference in expression level between fruit samples of ‘AF’ or ‘BSKP’ at 30 and 90 DAFB respectively. A total of 21 909 genes were consistently expressed in fruits of both ‘AF’ and ‘BSKP’ at the two stages tested. These consistently expressed genes shared 77 common genes (Table S2) with the common down‐regulated genes mentioned above, which contain putative candidate genes for fruit acidity.

Subsequently, functional annotation was conducted for these 77 genes based on Arabidopsis thaliana Annotation Database TAIR (https://www.arabidopsis.org/). As a result, four genes (MDP0000193656, MDP0000287416, MDP0000280551 and MDP0000810883) with a potential role in controlling fruit acidity were identified. Among these genes, MDP0000193656 and MDP0000287416 were involved in organic acid metabolism, and encoded malic enzyme and pyruvate decarboxylase respectively. MDP0000280551 encoded pyrophosphate‐energized proton pump that was reported to participate in regulation of apoplastic pH and auxin transport (Khadilkar *et al*., 2016). MDP0000810883 encoded P‐type ATPase that is well known to mediate vacuole acidification. Since malate is the predominant organic acid in apple fruit and its accumulation is mainly driven by vacuolar storage (Etienne *et al*., 2013), MDP0000810883, designated *Ma10*, was deemed to be a strong candidate controlling fruit acidity in apple. The expression level of *Ma10* was approximately 10‐fold higher in fruits of ‘BSKP’ at both 30 and 90 DAFB than in fruits of ‘AF’ according to the DGE data (Figure 1c).

### Expression profiling of *Ma10* in apple fruits at different developmental stages

**Figure 2:**
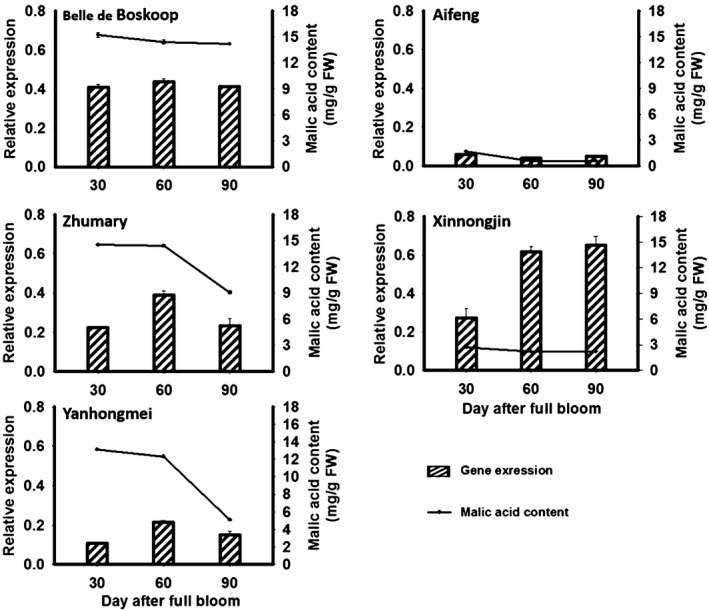
Expression profiling of *Ma10* gene and malic acid accumulation in apple fruit. All cultivars show similar ripening periods, with maturation occurring at approximately 90 days after full bloom. FW, fresh weight.

qRT‐PCR was conducted to investigate the expression profiles of *Ma10* in fruits of five cultivars, ‘Aifeng’, ‘Belle de Boskoop’, ‘Zhumary, Xinnongjin’, and ‘Yanhongmei’, at three developmental stages, with *ma1ma1*,* ma1ma1*,* Ma1ma1*,* Ma1ma1* and *Ma1 Ma1* in the *Ma* locus respectively (Figure 2). These five cultivars have similar ripening periods, with maturation occurring about 90 days after full bloom. Similar to the result of DGE mentioned above, the *Ma10* gene was consistently highly expressed in fruit of ‘BSKP’ at the three stages tested, whilst its transcript accumulation was extremely low in fruit of ‘AF’ throughout the developmental stages. In addition, the *Ma10* gene showed an increase in expression level in fruits of ‘Xinnongjin’ throughout the developmental stages. In contrast, the expression level of the *Ma10* gene showed a decrease in fruits of ‘Zhumary’ and ‘Yanhongmei’ at 90 DAFB, and had a peak in fruits at 60 DAFB.

We also investigated the correlation between transcript level of *Ma10* and malic acid content. Transcript accumulation patterns were very similar between ‘Yanhongmei’ and ‘Zhumary’, and malic acid contents were also similar. Similarly, a consistency between transcript level and malic acid content was observed in fruits of ‘AF’ and ‘BSKP’ throughout the developmental stages. For these four cultivars, the expression level of *Ma10* showed a consistency with malic acid content in fruits during the late developmental stages. However, although transcript level was higher in fruit of ‘Xinnongjin’ than in fruit of ‘BSKP’, at late developmental stages, ‘Xinnongjin’ had a very low level of malic acid accumulation in fruit throughout the developmental stages. This inconsistency could be due to the fact that genes other than *Ma10* are also involved in the regulation of malic acid accumulation in apple fruit.

### Gene family encoding P‐type ATPase in the apple genome

The amino acid sequence of the *Ma10* gene was BLASTed against the genome sequences of apple (Velasco *et al*., 2010), and fifteen homologs were identified on chromosomes 2, 4, 5, 6, 8, 9, 11, 13, 15 and 17 or among the unanchored sequences (Figure S2A). Phylogenetic analysis revealed that these homologs were divided into two gene families, designated P_3A_‐I and P_3A_‐II (Figure S2B). The P_3A_‐II family could be further divided into two subfamilies, P_3A_‐IIa and P_3A_‐IIb. The P_3A_‐I family consisted of two homologs, MDP0000303799 and MDP0000290422. The P_3A_‐IIa subfamily included seven homologs, MDP0000277881, MDP0000152620, MDP0000810883 (*Ma10*), MDP0000158160, MDP0000266132, MDP0000215211 and MDP0000157578, whereas, the P_3A_‐IIb subfamily contained seven homologs, MDP0000249645, MDP0000162032, MDP0000150049, MDP0000259837, MDP0000136397, MDP0000195785 and MDP0000181085. Moreover, the *P‐ATPase* genes showed a great variation in genomic structure, with the number of exons ranging from 14 to 22 (Figure S3). For example, MDP0000290422 comprised 22 exons spanning approximately 8.8 kb in physical length, whilst MDP0000249645 consisted of 14 exons spanning approximately 4.1 kb in size.

**Figure 3:**
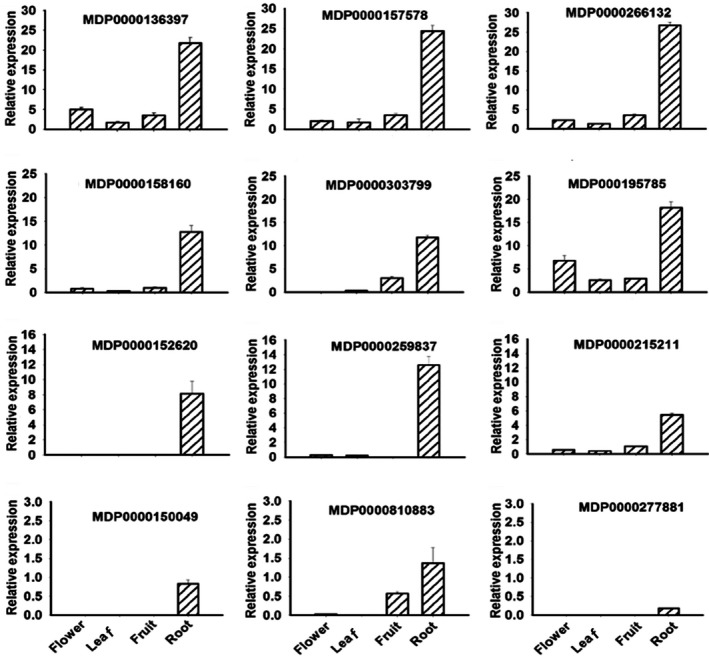
Expression profiling of the apple *P*_*3A*_ *‐ATPase* genes in four tissues, including full‐bloom flower, mature leaf, ripening fruit and root. The values represent the average of three biological replicates.

Expression profiles of the *P‐ATPase* genes were investigated in different tissues, including flower, leaf, root and mature fruit (Figure 3). Of these *P‐ATPase* genes, four were not expressed in any tissues tested, including MDP0000162032, MDP0000181085, MDP0000290422 and MDP0000249645. In contrast, six genes, MDP0000158160, MDP0000157578, MDP0000136397, MDP0000195785, MDP0000266132 and MDP0000215211 were expressed in all the tissues tested. Four genes, MDP0000152620, MDP0000277881, MDP0000150049 and MDP0000259837, were exclusively expressed in the root. The remaining two genes, MDP0000303799 and MDP0000810883 were mainly expressed in the root and fruit. Overall, the *P‐ATPase* genes showed relatively higher levels of expression in the root. It is worth noting that only the *Ma10* gene (MDP0000810883) out of the eight *P‐ATPase* genes that were expressed in fruit was found to be a candidate controlling fruit acidity based on comparative transcriptome analysis. Hence, the *Ma10* gene was further subjected to functional analysis.

### Association analysis between malic acid content and Ma10 in apple fruits

**Figure 4:**
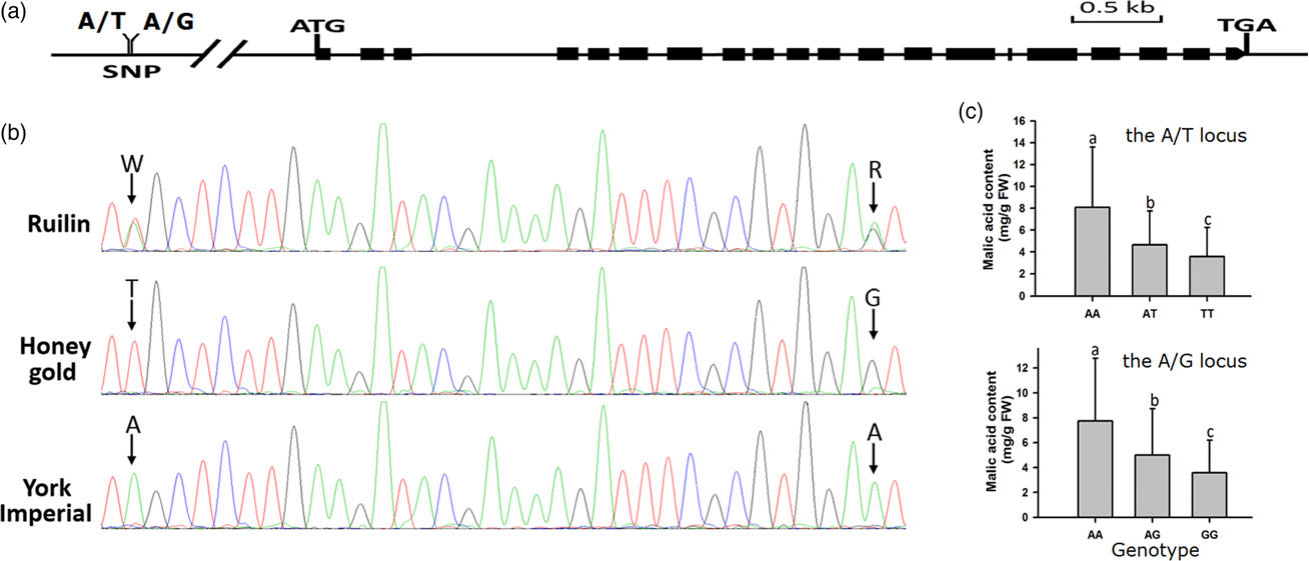
Characterization of the *Ma10* gene responsible for fruit acidity in apple. (a) Gene structure of the *Ma10* gene and two adjacent SNP loci, A/T and A/G, located 4328 and 4296 bp upstream of the start codon respectively. (b) An example of genotyping of the apple population using molecular tag of the *Ma10* gene. (c) Mean values of malic acid content in mature fruits of different genotypes that are classified according to the A/T and A/G SNPs of the *Ma10* gene in apple. Different lowercase letters indicate significant differences among genotypes (*t*‐test, LSD test at *P *<* *0.01). Error bars show the SE of the mean. FW, fresh weight.

Two adjacent polymorphic SNP loci, A/T and A/G, located 4328 and 4296 bp upstream of the *Ma10* translational start site were identified (Figure 4a) and subsequently used to screen a collection of 353 *Malus* accessions using PCR‐direct sequencing method (Figure 4b). For each SNP locus, two homozygous genotypes and one heterozygous genotype were identified. The two SNPs formed four haplotypes, T‐G, A‐A, A‐G and T‐A, with frequencies of 0.66, 0.31, 0.02 and 0.01 respectively. Candidate gene‐based association analysis showed that the A/T and A/G SNPs were both significantly associated with malic acid content, with *P‐*values of 1.63 × 10^‐5^ and 1.06 × 10^−4^, and accounted for 7.93% and 7.43% of the observed phenotypic variation respectively. Mean values of fruit malic acid content for A/A, A/T and T/T genotypes at the A/T locus were 8.08, 4.66 and 3.59 mg/g FW, while 7.74, 5.03 and 3.61 mg/g FW for A/A, A/G and G/G genotypes at the A/G locus respectively. Significant difference in malic acid content was observed among the three genotypes for both A/T and A/G SNP loci (Figure 4c). These results further confirm that the *Ma10* gene is a candidate controlling malic acid accumulation in apple fruit.

### Subcellular localization of Ma10 and its functional analysis through overexpression in yeast, tomato and apple callus

**Figure 5:**
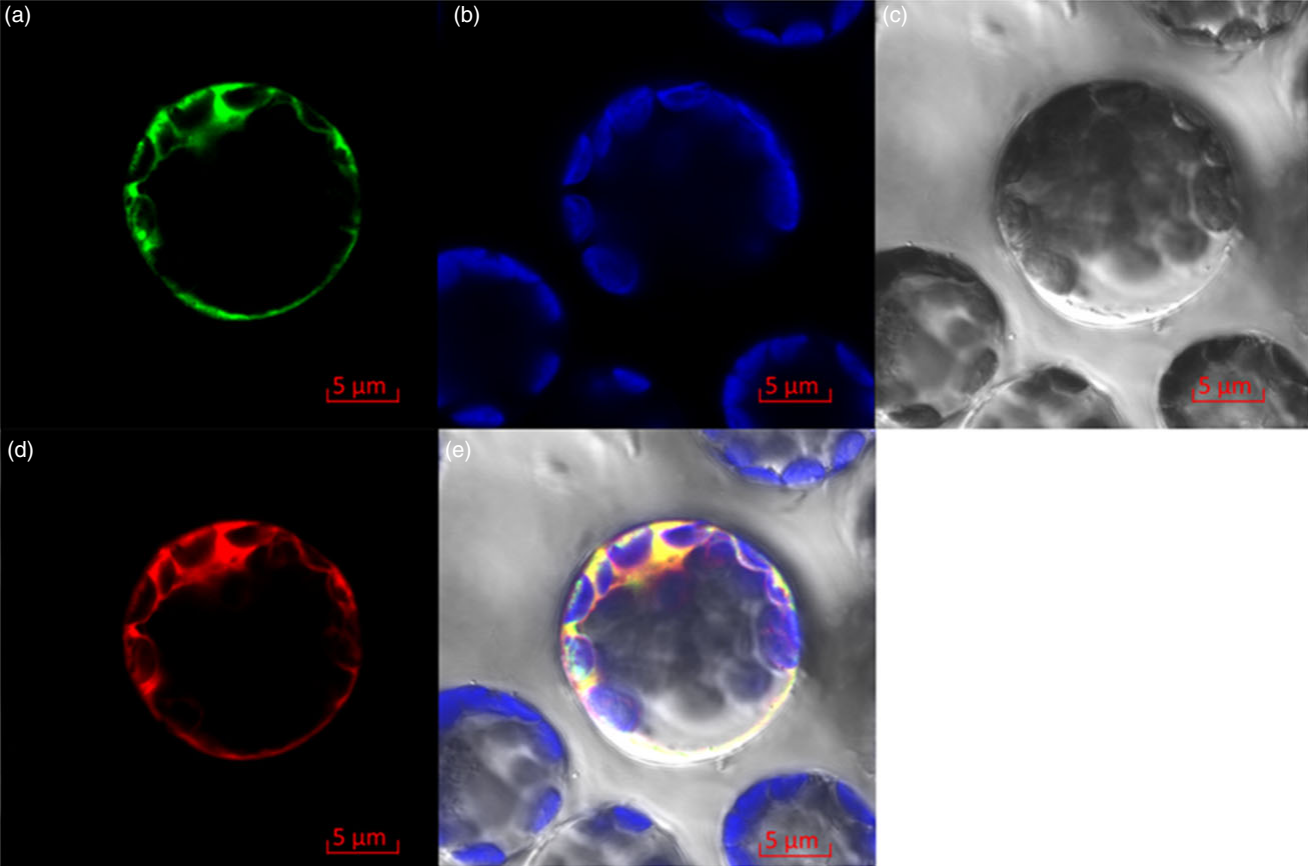
Subcellular localization of the Ma10‐GFP fusion protein in *Arabidopsis* mesophyll protoplast. (a) The fluorescence of Ma10‐GFP fusion protein; (b) Chloroplasts show auto fluorescence; (c) Optical photomicrographs (bright field); (d) The fluorescence of Vac‐rk (vacuolar marker) protein; (e) An overlay of bright and fluorescence illumination.

Similar to tonoplast P‐type ATPase proteins AtAHA10 and PhPH5, Ma10 contained four putative transmembrane domains (Figure S4). To further determine subcellular localization of Ma10, the Ma10‐GFP fusion construct under the control of CaMV 35S promoter was transiently expressed in *Arabidopsis* mesophyll protoplasts. It was also found that the Ma10‐GFP fusion protein resided in the tonoplast (Figure 5a,b,c). To clarify tonoplast location of Ma10, co‐localization was conducted using the standard vacuolar marker, Vac‐rk (CD3‐975) (Nelson *et al*., 2007; Figure 5d). The GFP fluorescence of Ma10‐GFP is completely merged with mCherry fluorescence of the tonoplast marker Vac‐rk (Figure 5e). Taken together, these results indicate that Ma10 is located in the tonoplast.

**Figure 6:**
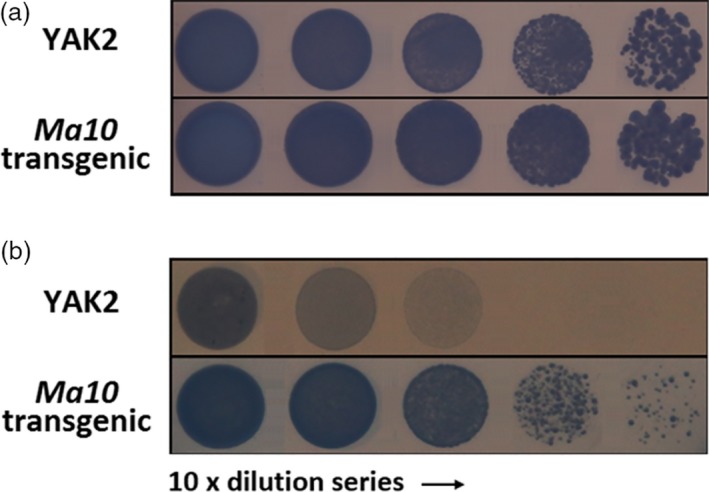
Complementation of the YAK2 yeast pma1/pma2 double‐mutant by a plasmid expressing Ma10. (a) yeast cell grown on acidic medium (pH 6.0) containing 2% galactose; (b) yeast cells grown on acidic medium (pH 6.0) containing 2% glucose.

To examine whether Ma10 has proton pumping activity, it was expressed in the YAK2 yeast strain, which has two disrupted plasma membrane H^+^‐ATPase genes, *PMA1* and *PMA2*, and contains a plasmid carrying the yeast *PMA1* gene under the control of a galactose‐inducible GAL1 promoter (de Kerchove d'Exaerde *et al*., 1995). Both the YAK2 strain and the transgenic strain expressing *Ma10* were able to grow on acidic medium containing 2% galactose (Figure 6a). However, YAK2 cells were unable to grow on acidic medium lacking galactose. By contrast, expression of *Ma10* sustained growth on non‐permissive medium (pH 6.0, 2% glucose) (Figure 6b), which suggests that Ma10 is able to complement the defect in proton transport of the YAK2 yeast strain. The ability to restore YAK2 growth on acidic media demonstrates that Ma10 has proton pumping activity. In addition, Ma10 rescued YAK2 cells less efficiently than yeast PMA1 (Figure 6). This might be due to the fact that the subcellular localization of plant tonoplast protein expressed in yeast is not only restricted to the vacuolar membrane, but also partially present on plasma membrane (Marinova *et al*., 2007; Verweij *et al*., 2008).

**Figure 7:**
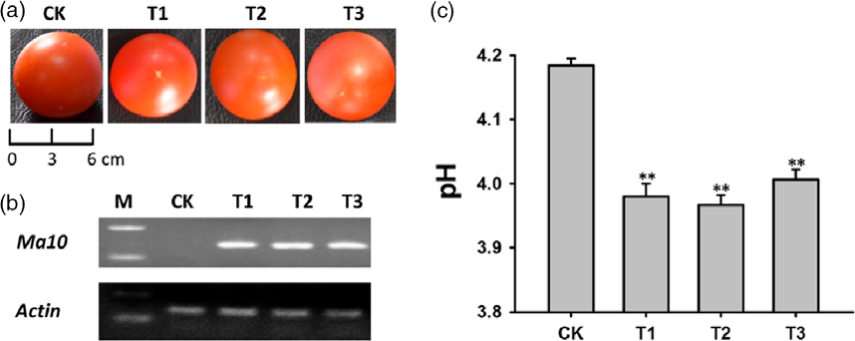
Functional analysis of the *Ma10* gene by ectopic expression in tomato. (a) Mature fruits of transgenic lines and wild type. (b) Expression analysis of *Ma10* in fruit using RT‐PCR. (c) The pH value in fruits of transgenic lines and wild type. CK indicates wild type, while T1, T2 and T3 represent transgenic lines. Double asterisks indicate significant differences between transgenic and wild‐type fruits (*t*‐test, *P *<* *0.01).

To further confirm whether overexpression of the *Ma10* gene has proton pumping activity, it was transformed into tomato. Three transgenic lines were generated and there was no significant difference in fruit size between transgenic lines and wild type (Figure 7a). Semi‐quantitative RT‐PCR analysis revealed that the *Ma10* gene was highly expressed in fruits of transgenic lines (Figure 7b). The pH values in transgenic and wild‐type fruits are shown in Figure 7c. The average pH value in transgenic fruits was 3.98, which was significantly lower than the pH value in wild‐type fruits (pH 4.18). Overall, transgenic fruits were significantly more acidic than wild‐type fruits. These results suggest that the *Ma10* gene plays an important role in organic acid accumulation in tomato fruit, leading to a decrease in the pH value.

**Figure 8:**
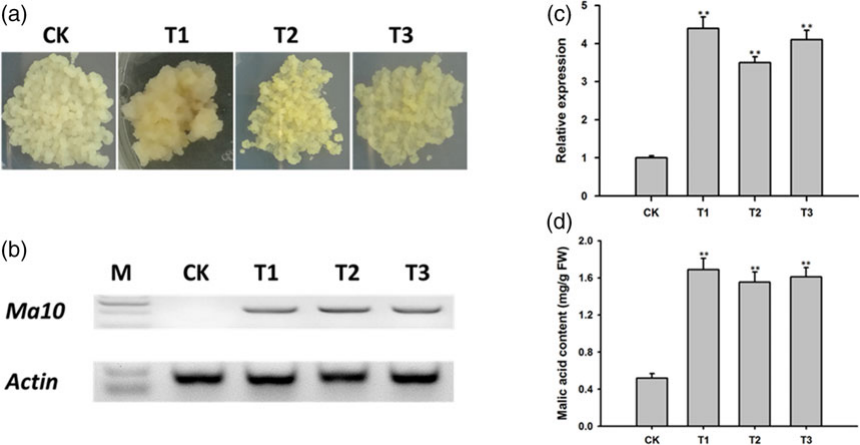
Functional characterization of *Ma10* through its overexpression in the apple callus of ‘Orin’. (a) Wild‐type and transgenic calli. (b) Validation of transgenic calli using PCR amplification. An 831‐bp fragment was amplified for transgenic calli, whilst no fragment for wild type as forward primer spanning two exons. (c) Expression analysis of *Ma10* in wild‐type and transgenic calli using qRT‐PCR. (d) Malic acid content in wild‐type and transgenic calli. CK indicates wild type, while T1, T2 and T3 represent transgenic lines. Double asterisks indicate significant differences between transgenic lines and wild type (*t‐*test, *P *<* *0.01).

To validate the function of *Ma10* gene in apple malic acid accumulation, *Ma10* was transformed into the callus of apple cv. Orin (Figure 8a). Three transgenic lines were generated and validated by PCR amplification (Figure 8b). Real‐time PCR assay clearly indicated that the transcript level of *Ma10* was significantly higher in transgenic lines than in the wild type (Figure 8c). Malic acid content in transgenic and wild‐type calli was measured and are shown in Figure 8d. The average malic acid content in transgenic apple calli was 1.61 mg/g FW, which was 3.10 times higher than that in wild‐type calli (0.52 mg/g FW). This result demonstrates that the *Ma10* gene is involved in malic acid accumulation in apple.

## Discussion

### *Ma10* gene encodes a tonoplast P_3A_ H^+^‐ATPase that is involved in the regulation of apple fruit acidity

In apple, fruit acidity is linked to the capacity to accumulate malic acid in vacuoles. The movement of malic acid from cytoplasm to vacuole is against an electrochemical potential gradient across the tonoplast (Δψ), which requires an energy‐dependent transport. This active transport is accomplished by two distinct types of proton pumps, V‐ATPase and V‐PPase, both of which are highly evolutionarily conserved throughout plant evolution (Kriegel *et al*., 2015; Lobit *et al*., 2006). V‐ATPase uses the energy from ATP hydrolysis to produce an electrochemical proton gradient, whilst V‐PPase uses inorganic pyrophosphate (PPi) as an energy source. These two types of proton pumps have been reported in a variety of fruit crops, such as apple (Hu *et al*., 2016), pear (Suzuki *et al*., 2000), grape (Terrier *et al*., 2001), and peach (Lobit *et al*., 2006). However, there have been few reports on the role of P‐type ATPase (P‐ATPase) in fruit acidity although P‐ATPases constitute a large superfamily of membrane proteins. The P‐ATPase superfamily can be divided into five subfamilies that translocate distinct cations (Axelsen and Palmgren, 1998).

In this study, we reveal that the P_3A_‐ATPase genes can be divided into two types, I and II. The apple genome has two type I P_3A_‐ATPase genes, which showed no expression in any tissues tested or no difference in expression between ‘AF’ and ‘BSKP’. Hence, both these two type I P_3A_‐ATPase genes are unlikely to be candidates for malate accumulation in apple fruit. By contrast, a type II P_3A_‐type ATPase gene, *Ma10*, was showed to be involved in the vacuolar acidification of apple fruit. P‐type ATPases are widely assumed to localize to the plasma membrane and use ATP as an energy source to secrete protons out of the cytoplasm into the apoplast (Gaxiola *et al*., 2007; Schumacher, 2014). However, the subcellular localization assay indicates that Ma10 resides in the tonoplast. This result is consistent with the recent finding that PH5 homologs evolved from plasma membrane P_3A_‐ATPases, but changed from the plasma membrane into tonoplast proteins due to an N‐terminal tonoplast‐sorting sequence acquisition before angiosperms appeared (Li *et al*., 2016b). Organic acid uptake is known to be driven by the electrical component of the electrochemical potential (Martinoia *et al*., 2000). Functional complementation of the YAK2 yeast strain suggests that the *Ma10* gene has the capacity for proton pumping, and its ectopic expression in tomato significantly increases organic acid accumulation in fruit. These results indicate that the Ma10 proton‐motive force is able to enhance the influx of protons, which not only reduces the vacuolar pH, but also provides a driving force for additional organic acid uptake. In addition, the *Ma10* gene is significantly associated with fruit malic acid content in apple germplasm. Taken together, all the above results indicate that the *Ma10* gene encodes a tonoplast P_3A_ H^+^‐ATPase controlling vacuolar acidification in apple fruit.

We compared the physical location between *Ma10* and previously reported QTLs for apple fruit acidity, and found that *Ma10* is physically close to the fruit acidity QTL on chromosome 17 (Kenis *et al*., 2008). This suggests that the *Ma10* gene could be the candidate gene within a QTL interval that is associated with apple fruit acidity. In addition, the P_3B_ H^+^‐ATPase PH1 can interact with the P_3A_ H^+^‐ATPase PH5 to boost PH5 H^+^ transport activity (Faraco *et al*., 2014). There are two PH1 homologs in the apple genome, MDP0000254558 and MDP0000319016, which are located on chromosomes 12 and 15 respectively (Li *et al*., 2016b). Interestingly, MDP0000319016 is located in the middle of chromosome 15 that harbours a QTL for titratable acidity (Kenis *et al*., 2008), which suggests that it is a good candidate for apple fruit acidity. It is worthy of further study to ascertain whether MDP0000319016 can physically interact with the *Ma10* gene to increase Ma10 H^+^ transport activity.

It is worth noting that five homologs of *Ma10* are located on chromosomes that harbour QTLs for fruit acidity. Of these homologs, two (MDP0000181085 and MDP0000152620) and one (MDP0000259837) are not expressed in all tissues tested or exclusively expressed in root. The remaining two, MDP0000195785 and MDP0000136397, are located on the top of chromosome 15, which is distant from the QTL interval controlling fruit acidity (Kenis *et al*., 2008). Therefore, these *Ma10* homologs are unlikely to be candidates for the QTLs controlling apple fruit acidity.

### Duplication and expression divergence of P_3A_‐ATPase genes in the apple genome

Gene duplication is a major driving force in evolution of genome and genetic systems and an important mechanism for the emergence of new genes and biological adaptation because over 90% of eukaryotic genes arise from gene duplication (Teichmann and Babu, 2004). Apple is a diploid, with an allopolyploid origin. Here, sixteen *P*_*3A*_ *‐ATPase* genes were identified in the apple genome. Of these genes, five are located on chromosomes 2, 8 and 15, with the two former chromosomes being both homologous to the latter chromosome (Han *et al*., 2011; Velasco *et al*., 2010). Similarly, three *P*_*3A*_ *‐ATPase* genes are located on homologous chromosome pairs, 9 and 17. In contrast, two tandems of *P*_*3A*_ *‐ATPase* genes were also found on chromosomes 6 and 15. These results suggest that duplication of *P*_*3A*_ *‐ATPase* genes is associated with both whole‐genome and segmental duplications.

To reveal the potential relationship between gene duplication and expression divergence, we compared the expression profiles of the duplicated *P*_*3A*_ *‐ATPase* genes mentioned above. As shown by qRT‐PCR, four genes, MDP0000162032, MDP0000181085, MDP0000290422 and MDP0000249645, showed no expression in any tissues tested. Interestingly, the first three genes arise from whole genome duplication. However, four genes that form two tandem pairs, MDP0000215211/MDP0000157578 and MDP0000136397/MDP0000195785, on chromosomes 6 and 15 respectively, were all expressed in any tested tissues. These results indicate that gene copies encoding P_3A_‐ATPases tend to be functional when generated by segmental duplication, but prone to be silenced when derived from polyploidization. These results are consistent with previous findings that gene duplication increases expression diversity (Li *et al*., 2005), and is accompanied by the preferential retention of some duplicates (Panchy *et al*., 2016). However, more studies are still needed to clarify whether this gene expression divergence is caused by subfunctionalization and/or neofunctionalization after gene duplication.

### Genetic complexity of organic acid accumulation in apple fruit

Apple fruit acidity is a quantitative trait and genetic mapping studies have revealed at least nine QTLs for fruit acidity in apple. However, only one candidate gene for fruit acidity, termed *Ma1*, was identified within the QTL interval on LG16 and functions as a malate channel to facilitate malate uptake into the vacuole (Bai *et al*., 2012; Ma *et al*., 2015b). Although *Ma1* is a major gene for fruit acidity, its mutation does not result in low content of organic acids in fruit of certain cultivars such as Belle de Boskoop (Ma *et al*., 2015b). Here, we show the *Ma10* gene encoding P_3A_‐ATPase, which functions as a proton pump to regulate vacuolar acidification of apple fruit. Thus, there are at least two distinct malate translocation systems, a vacuolar ALMT channel and a P_3A_‐ATPase proton pump, which are crucial for malate uptake into the vacuole in apple. It is worth noting that *Ma10* is highly expressed in fruit of ‘Xinnongjin’ during late developmental stages. Our previous study also showed that *Ma1* had an increase in expression throughout fruit development of ‘Xinnongjin’, and reached a relative high level at 90 DAFB (Ma *et al*., 2015b). However, ‘Xinnongjin’ showed very low levels of malic acid accumulation in fruit throughout the developmental stages. This result suggests that there are other genes besides *Ma1* and *Ma10* that control malate accumulation in apple fruit.

**Figure 9:**
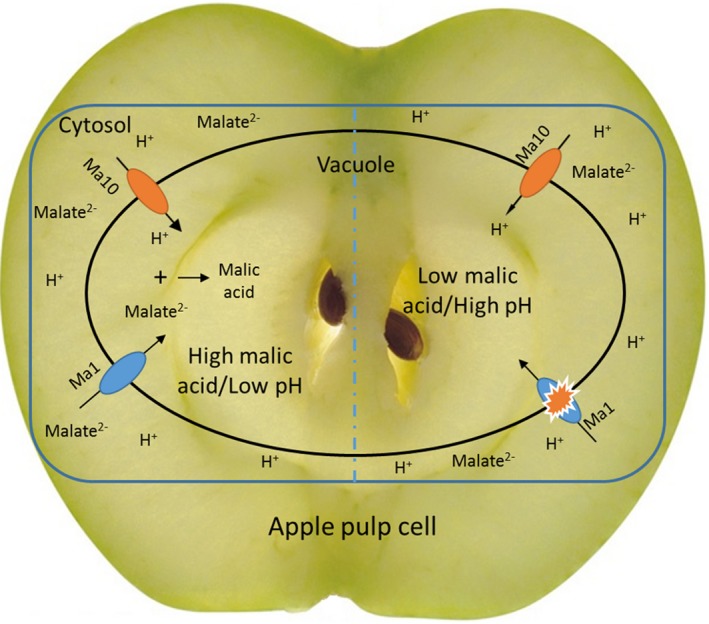
A proposed working model of malic acid accumulation in apple fruit. Ma10 pumps protons from the cytosol to the vacuole, while Ma1 mediates transport of malate from the cytosol to the vacuole. High expression of the *Ma10* allele with the thicker arrow is prone to cause an electrochemical gradient across the vacuole membrane, which induces transport of malate into the vacuole through the Ma1 channel. Low expression of the *Ma10* allele with the thinner arrow is insufficient to activate the Ma1 channel, resulting in low level of malate accumulation.

The *Ma10* gene is associated with fruit malate content in apple germplasm. According to the A/T or A/G SNPs in the promoter of *Ma10*, three genotypes were identified in all the apple accessions tested. The mean values of malic acid content in mature fruits showed significant differences among the three genotypes and displayed the following patterns: AA > AT > TT or AA > AG > GG. Previous studies reveal three genotypes in the *Ma1* locus, *Ma1 Ma1*,* Ma1ma1* and *ma1ma1*, based on an A/G SNP 252 bp upstream of the stop codon of the *Ma1* gene. All the apple accessions tested in this study can be divided into nine genotypes based on the presence of *Ma1* and *Ma10* alleles. When the *Ma10* locus is A/A at the A/T or A/G loci, the dominant homozygous *Ma1 Ma1* genotype had the highest fruit malate content (Table S3). This suggests the possibility of an interaction between the Ma10 proton pump and the Ma1 transport channel. To address this hypothesis, the expression level of *Ma1* was also evaluated in transgenic and wild‐type apple calli. Similar to *Ma10*,* Ma1* showed significantly higher expression in transgenic calli than in wild‐type calli (Figure S5). This suggests that transport of protons into the vacuole by Ma10 enhances the electrochemical gradient across the vacuole membrane, which in turn activates the Ma1 channel to increase the malate uptake into the vacuole. Once the malate dianion is transported into acidic vacuole, they immediately become protonated and accumulate in the vacuole. Thus, we propose a model of malic acid accumulation through the interaction between the Ma10 and Ma1 proteins in apple (Figure 9). Low expression of *Ma10* is unfavourable to activate the Ma1 channel to induce malate uptake into the vacuole.

In addition, evidence for interaction between proton pump genes has been reported. For example, P_3B_‐ATPase is able to interact with P_3A_‐ATPase to increase vacuolar acidification (Eisenach *et al*., 2014; Faraco *et al*., 2014). The combined activity of V‐ATPase and V‐PPase is required for vacuolar acidification, and the presence of the V‐PPase is crucial for increase in V‐ATPase activity during cold acclimation (Kriegel *et al*., 2015). In addition, transcription factors, such as WRKY, ERF and MYB genes, have been shown to interact with proton pump genes (Hu *et al*., 2016; Li *et al*., 2016a; Quattrocchio *et al*., 2006; Verweij *et al*., 2016). Hence, fruit vacuolar acidification appears to be genetically complex and probably regulated by complex gene regulation networks.

## Material and methods

### Plant materials

The population of apple germplasm used in this study is maintained at the Xingcheng Institute of Pomology of the Chinese Academy of Agricultural Sciences, Xingcheng, Liaoning, China. The population consisted of the 353 apple accessions as reported in our previous study (Ma *et al*., 2015a), and their leaf DNA samples together with database of the malic acid content in mature fruits were used for candidate gene‐based association mapping in this study. In addition, five cultivars, ‘Belle de Boskoop’, ‘Aifeng’, ‘Zhumary’, ‘Xinnongjin’ and ‘Yanhongmei’, were selected for RT‐PCR assay and/or transcriptome sequencing. Fruit samples were collected at 30, 60 and 90 days after full bloom (DAFB). Fruit samples were peeled, and pulps were cut into small pieces, immediately frozen in liquid nitrogen and then stored at −75 °C for later use.

### RNA‐seq library construction and sequencing

A total of 3 μg RNA per sample were subjected to RNA‐seq library construction using NEBNext^®^ Ultra™ RNA Library Prep Kit for Illumina^®^ (NEB, USA) according to the manufacturer's instructions. Briefly, the poly‐T oligo‐attached magnetic beads were used to extract mRNA molecules from total RNA, and the purified mRNA was fragmented using divalent cations under elevated temperature in NEBNext First Strand Synthesis Reaction Buffer (5×). The random hexamer primer and M‐MuLV reverse transcriptase (RNase H^−^) were used to synthesize the first strand cDNA, and the second strand cDNA was subsequently synthesized using DNA Polymerase I and RNase H. The overhangs were converted into blunt ends using exonuclease/polymerase. Following adenylation of 3′ ends of cDNA fragments, NEBNext Adaptor with hairpin loop structure were ligated to prepare for hybridization. The fragments with 150–200 bp in length were purified with AMPure XP system (Beckman Coulter, Beverly, USA), and then PCR amplification was performed to enrich the purified fragments. Finally, the PCR products were purified with the Agencourt AMPure XP kit, and library quality was evaluated using the Agilent Bioanalyzer 2100 system. A cBot cluster generation system was used to perform the clustering of the index‐coded samples based on TruSeq PE Cluster Kit v3‐cBot‐HS (Illumina) according to the manufacturer's instructions. The library sequencing was performed using the Illumina HiSeq 2000 platform (Illumina, San Diego, CA, USA) with 100‐bp paired‐end reads.

### Sequence analysis and mapping of clean reads

Raw data were filtered through in‐house Perl scripts, and clean reads were obtained by removing adapter sequences, empty reads and low quality sequences. The apple draft genome (Velasco *et al*., 2010) was downloaded from Genome Database for Rosaceae (https://www.rosaceae.org/). An index of the reference genome was built using Bowtie v2.0.6 (Langmead *et al*., 2009), and clean reads were aligned back to the apple draft genome using TopHat v2.0.9 (Trapnell *et al*., 2009).

### Differential gene expression analysis

Gene expression level was estimated using RPKM values (Reads Per Kilobase of exon model per Million mapped reads). HTSeq v0.5.4p3 was used to count the number of clean reads that were mapped to each gene. The read counts of each sequenced library were standardized by scaling the number of reads in a given library to a common value using the program edgeR version 2.6.10 (Robinson *et al*., 2010). Differential expression analysis was conducted using the DEGSeq R package (1.12.0; TNLIST, Beijing, China). The *P*‐values were adjusted based on the method described by Benjamini and Hochberg (1995), and *Q*‐value of 0.005 and log2‐fold change of 1 were set as the threshold for significantly differential gene expression. Differentially expressed genes were compared against NCBI RefSeq nucleotide database, UniPro protein and Swiss‐Prot databases, and then annotated based on the BLAST results, followed by the pathway annotation pipelines, including KEGG (www.genome.jp/kegg/) and GO (http://www.geneontology.org).

### RNA isolation and quantitative real‐time RT‐PCR (qRT‐PCR) analysis

Total RNA was isolated using the RNAprep pure Plant kit (Tiangen, Beijing, China) according to the manufacturer's instructions, and approximately 3 μg of total RNA per sample was used for cDNA synthesis using TransScript One‐Step gDNA Removal and cDNA Synthesis SuperMix (TRANS, Beijing, China) following the manufacturer's instructions. A SYBR Green‐based real‐time PCR assay was performed in a total volume of 20 μL reaction mixture containing 10 μL of 2× SYBR Green I Master Mix (Takara, Dalian, China), 0.2 μm of each primer and 100 ng of template cDNA. All the qRT‐PCR amplifications were performed using Applied Biosystems 7500 Real‐Time PCR Systems (Applied Biosystems, Foster City, CA). Melting curve analysis was performed at the end of 40 cycles to identify specific amplification products. An actin gene described in a previous study (Ma *et al*., 2015b) was used as a constitutive control. Negative control was also run for each sample, and each treatment consisted of three biological replicates. Primer sequences for qRT‐PCR analysis are listed in Table S1.

### Phylogenetic and statistical analyses

Amino acid sequences were aligned using CLUSTAL X. The resulting data matrix was subjected to phylogenetic tree analysis with MEGA software version 6 using Neighbor‐Joining (NJ) method, with the following parameters: bootstrap, 5000 replicates; model, p‐distance; and gaps/missing data treatment, pairwise deletion. All statistical analyses were carried out using SPSS statistics 17.0 (SPSS Inc., Chicago, Illinois), and significant difference values were estimated using two‐tailed test.

### Development of molecular tag of Ma10 and marker‐trait association

Promoter sequences of *Ma10* gene were retrieved from the draft genome of ‘Golden Delicious’ (Velasco *et al*., 2010) to design PCR primers to amplify ‘Belle de Boskoop’ and PCR products were sequenced using forward primer. Single nucleotide polymorphisms (SNPs) with primer sequences of 5′‐ATGAATGTTCTCCGTTAC‐3′/5′‐TTAGATATGATCCCACCT‐3′ were successfully developed to screen apple germplasm. Candidate gene‐based association mapping was performed with the software package TASSEL version 5.0 using a mixed linear model (Bradbury *et al*., 2007). The apple germplasm collection and population structure such as the Q matrix and the K matrix were the same as described by Ma *et al*. (2015b). Candidate gene association analysis was performed using a mixed linear model (MLM). The criterion for marker–trait association was set at *P *≤* *0.05 according to the false discovery rate (Benjamini and Yekutieli, 2001).

### Subcellular localization of Ma10 in Arabidopsis protoplast

The entire coding region of *Ma10* was amplified from cDNA templates in fruits of ‘Belle de Boskoop’ using a pair of primers, 5′‐CGATAGGCAGAGATGGATGAGA‐3′/5′‐TAACAGTGTATGCTTGCTGGA‐3′. RT‐PCR products were purified and inserted into the TA cloning vector pEASY‐T1. Later, the full CDS of *Ma10* was amplified and inserted into pHBT‐GFP‐NOS vector under the control of the cauliflower mosaic virus (CaMV) 35S promoter using a pair of primers, 5′‐GCTTCGAATTCTGCAGTCGACATGGATGAGAAAGCTGTCG‐3′ and 5′‐GCCCTTGCTCACTACGGATCCAACAGTGTATGCTTGCTGGA‐3′, which added *Sal*I and *Bam*HI sites at the 5′ and 3′ ends respectively. As a result, a construct constitutively expressing the fusion protein Ma10‐GFP (fluorescent protein) was generated and co‐transformed with the vacuolar membrane marker (vac‐rk CD3‐975) that contained the mCherry reporter gene (Nelson *et al*., 2007). PEG‐calcium transfection was conducted using *Arabidopsis* mesophyll protoplasts, which were isolated from well‐expanded leaves of 3–4 week old seedlings using the protocol described by Yoo *et al*. (2007). Detection of green fluorescent protein in *Arabidopsis* protoplast was performed at 12–24 h after transfection using a confocal laser scanning microscope Leica TCS SP8 (Leica, Germany). Different filter settings used to measure the GFP and mCherry fluorescence and chlorophyll autofluorescence were 488, 651 and 750 nm, respectively.

### Complementation analysis of the yeast mutant YAK2

Yeast expression vector pGADT7 AD was digested with HindIII and then ligated with a multiple cloning site (MCS) from pUC19 vector, generating a new yeast expression vector *H401*. The full coding region of *Ma10* was amplified from fruit cDNAs of ‘Belle de Boskoop’ using a pair of primers, 5′‐GCGCTCTAGACGATAGGCAGAGATGGATGAGA‐3′ and 5′‐CCGGGGATCCGAGACTTGGATACTCGGAGACTC‐3′, which added *Xba*I and *Bam*HI sites at the 5′ and 3′ ends respectively. PCR products were digested with *Xba*I and *Bam*HI and then inserted into the *H401* vector under the control of the ADH1 promoter, generating a construct *H401‐Ma10*. Subsequently, *H401‐Ma10* was transformed to YAK2 cells using the polyethylene glycol–lithium acetate method (Gietz and Woods, 2002), and the transformed cells were grown on the plates with pH 6.0 containing 2% galactose. The YAK2 cell was provided by Dr. Francesca M. Quattrocchio in VU University, Amsterdam, Netherlands. To avoid any false positives, positive colonies were confirmed again using the PCR sequencing approach.

### Functional analysis of Ma10 in transgenic tomato and apple callus

A pair of primers, 5′‐GCGGTTAATTAACGATAGGCAGAGATGGATGAGA‐3′ and 5′‐CGGCGGCGCGCCGAGACTTGGATACTCGGAGACTC‐3′, which added *Pac*I and *Not*I sites at the 5′ and 3′ ends respectively, was designed to amplify the whole coding sequence of *Ma10* using cDNA templates from fruit of ‘Belle de Boskoop’. PCR products were digested with *Pac*I and *Not*I and then inserted into the expression vector pMDC83, generating a construct pMDC83‐Ma10. The pMDC83‐Ma10 construct was introduced into *Agrobacterium tumefaciens* strain GV3101 by electroporation, and the nucleotide sequence of inserted fragments were validated by the PCR sequencing method.

The pMDC83‐Ma10 construct was transformed into tomato (Lycopersicon esculentum Mill. cv. Money Maker) using the Agrobacterium‐mediated leaf disc infiltration method (Frary and Earle, 1996). Transformants were selected on MS medium containing 50 μg/mL of hygromycin B. The hygromycin‐resistant seedlings were transferred into soil and cultivated in greenhouse. Positive T_1_ plants were assayed again with the PCR sequencing method, and the expression of *Ma10* in fruit was validated using RT‐PCR (35 cycles) with the same primers as qRT‐PCR. Red fruit samples of transgenic plants were randomly harvested, and each transgenic line had three replicates, consisting of about 10 fruits. Fruit juice of each replicate was filtered with gauze and then transferred into 15 mL tubes. After centrifugation at 5000 ***g*** for 15 min, the supernatants were immediately used to measure fruit pH with a pH metre (DENVER INSTRUMENT, USA).

The pMDC83‐Ma10 construct was introduced into *Agrobacterium tumefaciens* strain LBA4404 to conduct apple callus transformation using the protocol described by Li *et al*. (2002). The transgenic apple calli were validated by PCR amplification using a pair of primers, 5′‐TTGCCAATGGAGGAGGAA‐3′/5′‐GAACAACAGTGTCTGCGTCC‐3′, with forward primer spanning exons 4 and 5, while reverse primer located in exon 10. Measurement of malic acid content in transgenic and wild‐type calli was conducted following the procedure described by Ma *et al*. (2015a). Briefly, approximately 0.1 g of calli was ground into powder in liquid nitrogen, homogenized with 1.0 mL ddH_2_O and incubated at room temperature for 5 min. The mixture was then centrifuged at 12 000 rpm for 15 min at 4 °C. The supernatant was passed through a SEP‐C18 cartridge (Supel clean ENVI C18 SPE), and filtered through a 0.45 μm Sep‐Pak filter. The supernatants were subjected to measure malic acid content using high performance liquid chromatography. Organic acid concentration was expressed in mg/g FW (fresh weight).

## Conflict of interest

The authors declare no conflict of interest, in accordance with the policy described in the Instructions for Author (http://onlinelibrary.wiley.com/journal/10.1111/%28ISSN%291467-7652/homepage/ForAuthors.html).

## Author contributions

Y.H. and B.M. conceived and designed the experiments. B.M., L.L., T.F., Q.P. and H.Z. performed the experiments. B.M. and H.Z. wrote the paper. C.O. revised the manuscript.

## Supporting information


**Figure S1** Cluster analysis of expression profiles of genes that show differential expression between any two fruit samples.
**Figure S2** Genes encoding P_3A_‐ATPase in the apple genome.
**Figure S3** Schematic overview of genomic structure of sixteen *Ma10* homologs in apple. The black box and single line indicate exon and intron respectively.
**Figure S4** Alignment of amino acid sequences of Ma10 and its two homologs, AtAHA10 in *Arabidopsis* and PhPH5 in petunia. Four putative transmembrane domains that were identified in the N‐terminal region using TMHMM Server v. 2.0 (http://www.cbs.dtu.dk/services/TMHMM-2.0/) were highlighted in black background.
**Figure S5** Analysis of gene expression of *Ma1* in apple calli using qRT‐PCR. CK indicates wild type, while T1, T2 and T3 represent transgenic lines. Asterisks indicate significant differences between transgenic lines and wild type (*t*‐test, *P *<* *0.01).
**Table S1** Primers used for qRT‐PCR analysis in apple.
**Table S2** The 77 common down‐regulated genes in apple cultivars AF and BSKP.
**Table S3** Mean values of malic acid contents (mg/g FW) in mature fruits of different genotypes that are classified according to the A/T SNP of the *Ma10* gene and the A/G SNP of the *Ma1* gene in apple.
