# Supplementary material for: A Ma10 gene encoding P‐type ATPase is involved in fruit organic acid accumulation in apple
Source: Plant Biotechnol J. 2018 Nov 1;17(3):674–86. doi: 10.1111/pbi.13007 (PMC6381788; doi:10.1111/pbi.13007)
Supplement: Supplementary file 1 — Figure S1 Cluster analysis of expression profiles of genes that show differential expression between any two fruit samples. Figure S2 Genes encoding P3A‐ATPase in the apple genome. Figure S3 Schematic overview of genomic structure of sixteen Ma10 homologs in apple. The black box and single line indicate exon and intron respectively. Figure S4 Alignment of amino acid sequences of Ma10 and its two homologs, AtAHA10 in Arabidopsis and PhPH5 in petunia. Four putative transmembrane domains that were identified in the N‐terminal region using TMHMM Server v. 2.0 (http://www.cbs.dtu.dk/services/TMHMM-2.0/) were highlighted in black background. Figure S5 Analysis of gene expression of Ma1 in apple calli using qRT‐PCR. CK indicates wild type, while T1, T2 and T3 represent transgenic lines. Asterisks indicate significant differences between transgenic lines and wild type (t‐test, P < 0.01). Table S1 Primers used for qRT‐PCR analysis in apple. Table S2 The 77 common down‐regulated genes in apple cultivars AF and BSKP. Table S3 Mean values of malic acid contents (mg/g FW) in mature fruits of different genotypes that are classified according to the A/T SNP of the Ma10 gene and the A/G SNP of the Ma1 gene in apple. [file PBI-17-674-s001.docx]

Supplemental data

Table S1 Primers used for qRT-PCR analysis in apple

| Gene ID | Forward primer(5’→3’) | Reverse primer(5’→3’) |
| --- | --- | --- |
| MDP0000810883 | CGGAGCAGATTTTGAACCTT | GGGTCGAACAGAGGCAAGA |
| MDP0000136397 | TGACTGGAGAAGAAGGAGCC | GCAATAGCCATCAGAGCAGC |
| MDP0000150049 | AATGGAGTGAGCAGGATGCT | CACAAGATGTGCAGCCTTCC |
| MDP0000152620 | GACGCAATCATCAAGGAAGC | TGACCCAGGACAGAGGATTC |
| MDP0000157578 | GCCTGAAGTATTGGATGCTG | CCCATGAGAGAGGATTCCAC |
| MDP0000158160 | ATGGGGGAGAAACCTGAAGT | CCATGAGAGAGGATTCCACA |
| MDP0000162032 | GAGGAAGTGTTCGAGCAGCT | AGTCAGTTGGCTTGCCCA |
| MDP0000181085 | CGTCACTGATTCCCACCACT | ATCTCTTCGAGGCTGATGGC |
| MDP0000195785 | GCAAGTACAGGCAAGGGATT | TCAGGGTCAAAGTCCCAGTC |
| MDP0000215211 | GCCTGAAGTATTGGATGCTG | GAGGATTCCACATAAACCCC |
| MDP0000249645 | TCGGACCTTGTTTAATGGCT | GGATTCCACATGAAACCCAA |
| MDP0000259837 | AAGAAAACAATGCCGGAAA | GTGATTCTCCTGTGAGGGCA |
| MDP0000266132 | TGGATGCTGTGTTGAAGGAA | TGCTCTCCTGCTTTTCTTCA |
| MDP0000277881 | TGGGATTTATGTGGAACCCT | GGAGCCAGTCTTGCCATAAG |
| MDP0000290422 | AGTGTCATCGTTGGAAAACG | CCCATTTTCAACCTAACGGT |
| MDP0000303799 | CCATTGTTGGATCCTGAAAA | CATGACAAGGGGTTCCACAT |
| MDP0000146795 | GGTCTTCTCCCCCTATTTGATCCAC | CCCATCCCAAGTCGTCTCCCAGT |
| MDP0000278361 | CCCCTATTTGATCCACCACGTC | CCCAAGTCGTCTCCCAGTTTCTTT |
| Actin | TGACCGAATGAGCAAGGAAATTACT | TACTCAGCTTTGGCAATCCACATC |

Table S2 The 77 common down-regulated genes in apple cultivars AF and BSKP

| Gene ID | Chr. | Location (Mb) | Gene annotation |
| --- | --- | --- | --- |
| MDP0000215166 | 0 | 76.5 | Adenine nucleotide alpha hydrolases-like superfamily protein |
| MDP0000329265 | 0 | 92.6 | Ribosomal protein S30 family protein |
| MDP0000602911 | 0 | 78.7 | Calcium-binding EF hand family protein |
| MDP0000836404 | 0 | 3.2 | Oligosaccharyltransferase complex/magnesium transporter family protein |
| Novel05206 | 0 | 6.4 | MDC021421.104 |
| MDP0000152574 | 1 | 25.5 | 25.3 kDa vesicle transport protein-like |
| MDP0000252267 | 1 | 23.8 | PLAC8 family protein |
| MDP0000615948 | 1 | 28.6 | Helix-turn-helix motif/Leucine zipper, homeobox-associated |
| MDP0000940313 | 1 | 27.5 | ATCHIA, CHIA \| chitinase A \| |
| Novel00294 | 1 | 25.2 | MDC001207.483 |
| Novel05338 | 1 | 21.8 | MDC022074.32 |
| MDP0000157238 | 2 | 11.4 | Gibberellin regulated protein |
| MDP0000193656 | 2 | 24.6 | Malic oxidoreductase/Malic enzyme, NAD-binding |
| MDP0000288619 | 2 | 13 | Unknow protein |
| MDP0000340542 | 2 | 23.9 | Calcium-binding EF-hand/EF-hand-like domain |
| MDP0000776958 | 2 | 28.8 | glyceraldehyde-3-phosphate dehydrogenase C subunit 1 |
| MDP0000941000 | 2 | 10.6 | Amino acid transporter, transmembrane |
| MDP0000096349 | 3 | 32.3 | glutathione S-transferase |
| MDP0000140046 | 3 | 3.1 | farnesyl diphosphate synthase 1 |
| MDP0000165136 | 3 | 11.4 | ABC transporter family protein |
| MDP0000266097 | 3 | 32.3 | Dephospho-CoA kinase/Glutathione S-transferase/Thioredoxin-like fold |
| MDP0000363054 | 3 | 6.3 | Cyclophilin-like peptidyl-prolyl cis-trans isomerase domain |
| MDP0000492839 | 3 | 6.9 | Tubulin |
| MDP0000690963 | 3 | 5.8 | Pleckstrin homology (PH) domain superfamily protein |
| MDP0000929994 | 3 | 31.8 | Lipase, GDXG, active site/Alpha/beta hydrolase fold-3 |
| MDP0000120294 | 4 | 19.9 | Calcium-binding EF-hand family protein |
| MDP0000253535 | 4 | 2.8 | cysteine-rich RLK (RECEPTOR-like protein kinase) 10 |
| MDP0000306494 | 4 | 10 | PLAC8 family protein |
| MDP0000181169 | 5 | 15.4 | Thiamine pyrophosphate dependent pyruvate decarboxylase family protein |
| MDP0000223243 | 5 | 15.4 | Thiamine pyrophosphate dependent pyruvate decarboxylase family protein |
| MDP0000280551 | 5 | 19.3 | Pyrophosphate-energised proton pump |
| MDP0000362286 | 5 | 4.6 | dormancy-associated protein-like 1 / DRM1, DYL1 \| dormancy-associated protein-like 1 |
| MDP0000641053 | 5 | 30 | Unknow protein |
| MDP0000203894 | 6 | 1.7 | Zinc finger, RING/FYVE/PHD-type |
| Novel04180 | 6 | 24.9 | MDC017021.252 |
| MDP0000132058 | 8 | 29.2 | ATP binding; leucine-tRNA ligases; aminoacyl-tRNA ligases; nucleotide binding; ATP binding; aminoacyl-tRNA ligases |
| MDP0000267123 | 8 | 0.6 | senescence-associated gene 101 |
| MDP0000290516 | 8 | 28.9 | NIFU-like protein |
| MDP0000329640 | 8 | 22.3 | Ribosomal protein L7Ae/L30e/S12e/Gadd45 family protein |
| MDP0000541168 | 8 | 10 | actin cytoskeleton-regulatory complex protein PAN1-like |
| Novel04424 | 8 | 28.3 | MDC017993.238 |
| MDP0000738776 | 9 | 2.4 | WD40/YVTN repeat-like-containing domain |
| MDP0000161955 | 10 | 11.6 | squalene monooxygenase-like |
| MDP0000221381 | 10 | 5.6 | glycerol-3-phosphatase 1 |
| MDP0000279111 | 10 | 8.2 | FUNCTIONS IN: molecular_function unknown; INVOLVED IN: biological_process unknown; LOCATED IN: plasma membrane |
| MDP0000692555 | 10 | 14.7 | 2Fe-2S ferredoxin-like superfamily protein |
| MDP0000288468 | 11 | 34.4 | GAF domain/ATPase-like, ATP-binding domain/Signal transduction histidine kinase |
| MDP0000319522 | 11 | 33.7 | Lipase, GDXG, active site/Alpha/beta hydrolase fold-3 |
| MDP0000487255 | 11 | 11 | GTP binding Elongation factor Tu family protein |
| MDP0000689049 | 11 | 11.4 | Ribosomal protein L11 |
| MDP0000935996 | 11 | 5.2 | DNA-binding WRKY |
| Novel03323 | 11 | 2.7 | MDC012704.279 |
| MDP0000124144 | 12 | 26.5 | Protein of unknown function |
| MDP0000287416 | 12 | 24.6 | pyruvate decarboxylase-2 |
| MDP0000300444 | 12 | 30.1 | Aldo/keto reductase/NADP-dependent oxidoreductase domain |
| MDP0000425783 | 12 | 29.9 | Cysteine synthase/cystathionine beta-synthase P-phosphate-binding site/Pyridoxal phosphate-dependent enzyme, beta subunit |
| MDP0000709006 | 12 | 12.8 | Serine/threonine dehydratase, pyridoxal-phosphate-binding site |
| MDP0000786461 | 12 | 27.1 | Unknow protein |
| MDP0000293045 | 13 | 2.7 | Major facilitator superfamily protein/ ATPTR1, PTR1 \| peptide transporter 1 |
| MDP0000361768 | 13 | 14.4 | Unknow protein |
| MDP0000481448 | 13 | 4.3 | NAC transcription factor 29-like |
| MDP0000132720 | 14 | 17.7 | tubulin beta-6 chain-like |
| MDP0000190273 | 14 | 19.6 | Ethylene insensitive 3 family protein |
| MDP0000222447 | 14 | 1 | GroES-like zinc-binding alcohol dehydrogenase family protein/oxidoreductase, zinc-binding dehydrogenase family protein |
| MDP0000313341 | 14 | 4.5 | Ribosomal S17 family protein |
| MDP0000365732 | 14 | 17.7 | Unknow protein |
| MDP0000728753 | 14 | 7.6 | Leucine-rich repeat |
| MDP0000202144 | 15 | 5.7 | unknown protein |
| MDP0000269136 | 15 | 6.8 | vacuolar protein sorting-associated protein 20.2/SNF7 family protein |
| MDP0000383777 | 15 | 31.8 | Amino acid transporter, transmembrane |
| MDP0000397302 | 15 | 16.3 | MOB kinase activator-like 1 isoform X1 |
| MDP0000455885 | 15 | 46.3 | Unknow protein |
| MDP0000555175 | 15 | 16.9 | Aminotransferase, class V/Cysteine desulfurase |
| MDP0000161634 | 16 | 6.3 | INVOLVED IN: photorespiration; LOCATED IN: mitochondrial membrane, mitochondrial respiratory chain complex I, respiratory chain complex I |
| MDP0000240828 | 16 | 4.6 | ATP-grasp fold, succinyl-CoA synthetase-type |
| Novel03858 | 16 | 5.8 | MDC015361.139 |
| MDP0000810883 | 17 | 3.1 | ATPase, P-type, H+ transporting proton pump |

Table S3 Mean values of malic acid contents (mg/g FW) in mature fruits of different genotypes that are classified according to the A/T SNP of the *Ma10* gene and the A/G SNP of the *Ma1* gene in apple.

|  |  |  | *Ma1* | | |
| --- | --- | --- | --- | --- | --- |
|  |  |  | G/G | A/G | A/A |
| *Ma10* | The A/T locus | A/A | 13.11 | 7.71 | 4.54 |
|  |  | A/T | 6.23 | 5.06 | 4.20 |
|  |  | T/T | 4.39 | 4.24 | 3.00 |
|  | The A/G locus | A/A | 12.47 | 7.67 | 4.94 |
|  |  | A/G | 8.03 | 5.44 | 4.09 |
|  |  | G/G | 4.39 | 4.23 | 3.02 |


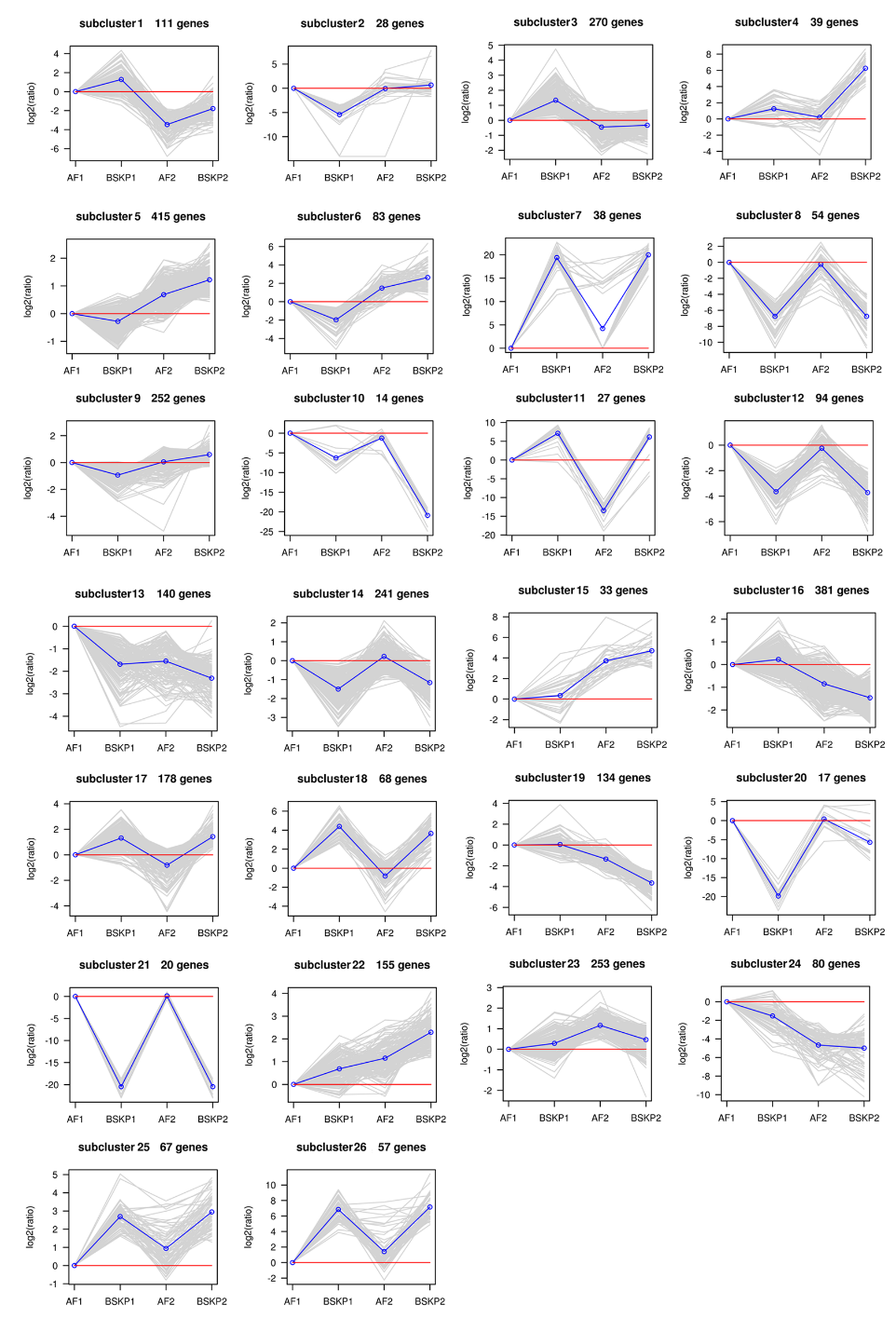


Fig. S1 Cluster analysis of expression profiles of genes that show differential expression between any two fruit samples.


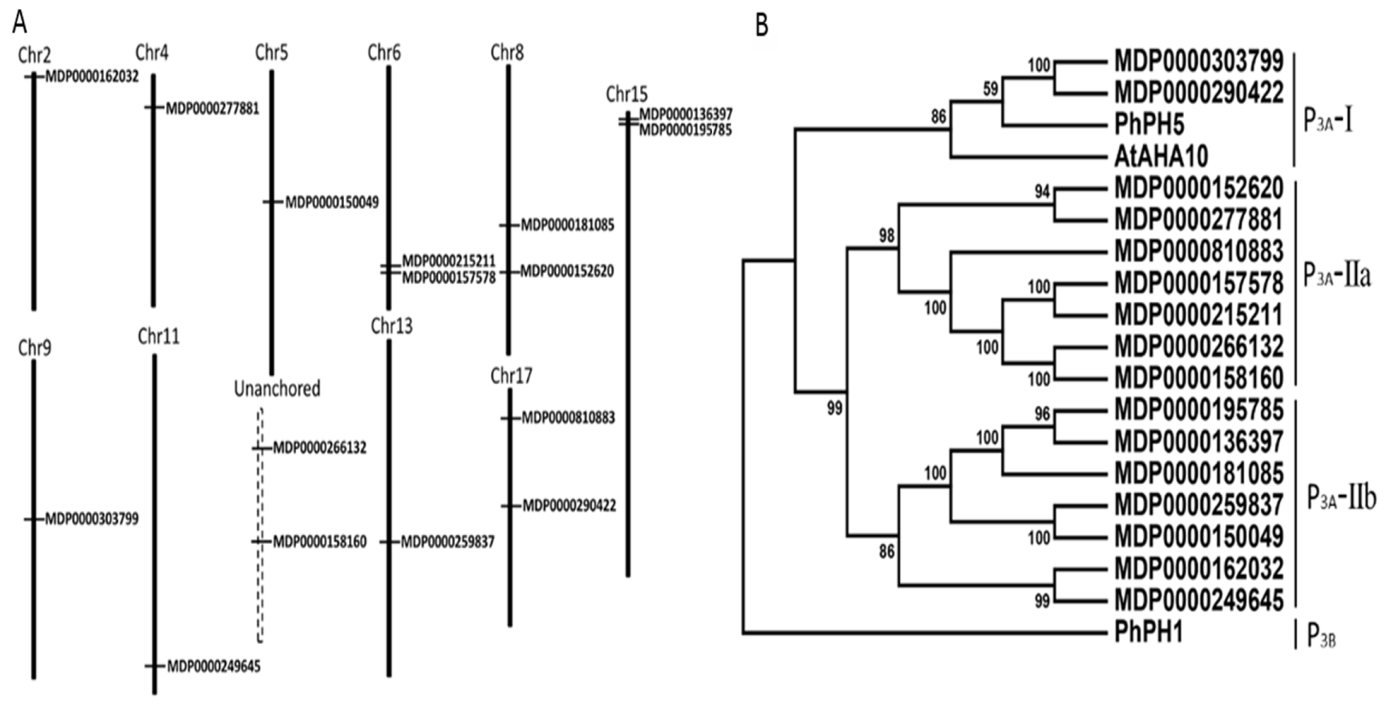


Fig. S2 Genes encoding P_3A_-ATPase in the apple genome. (A) Chromosomal distribution of the *P_3A_-ATPase* genes. The accession numbers are retrieved from the Genome Database for Rosaceae database (http://www.rosaceae.org). (B) Phylogenetic tree derived from amino acid sequences of the *P_3A_-ATPase* genes in apple and petunia.


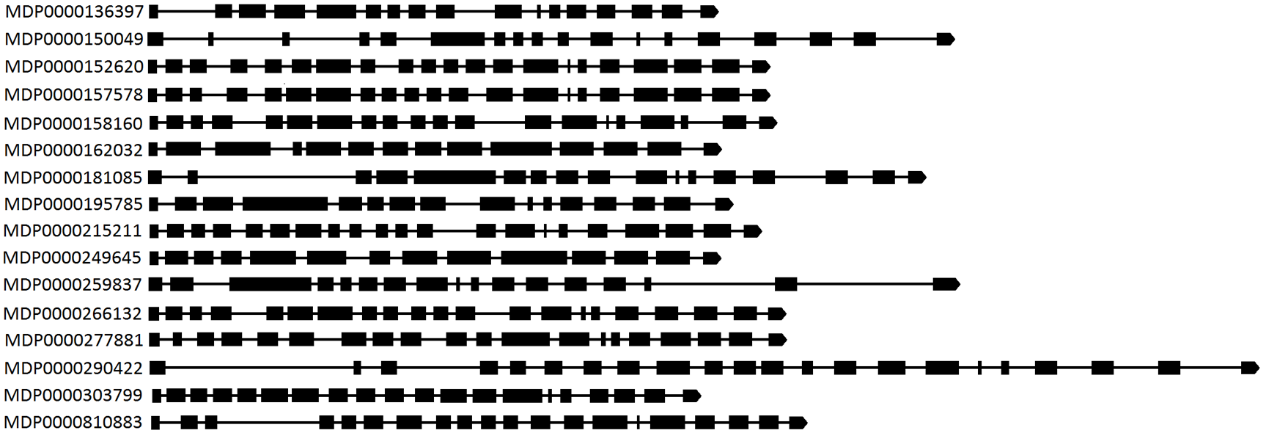


Fig. S3 Schematic overview of genomic structure of sixteen *Ma10* homologs in apple. The black box and single line indicate exon and intron, respectively.


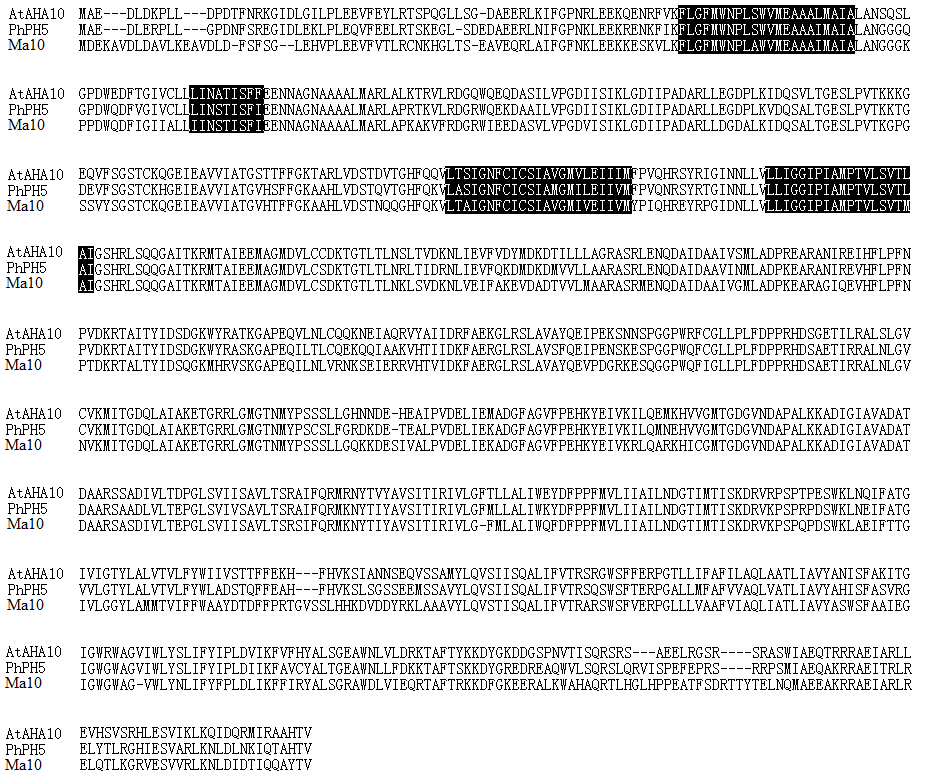


Fig. S4 Alignment of amino acid sequences of Ma10 and its two homologs, AtAHA10 in *Arabidopsis* and PhPH5 in petunia. Four putative transmembrane domains that were identified in the N-terminal region using TMHMM Server v. 2.0 (http://www.cbs.dtu.dk/services/TMHMM-2.0/) were highlighted in black background.


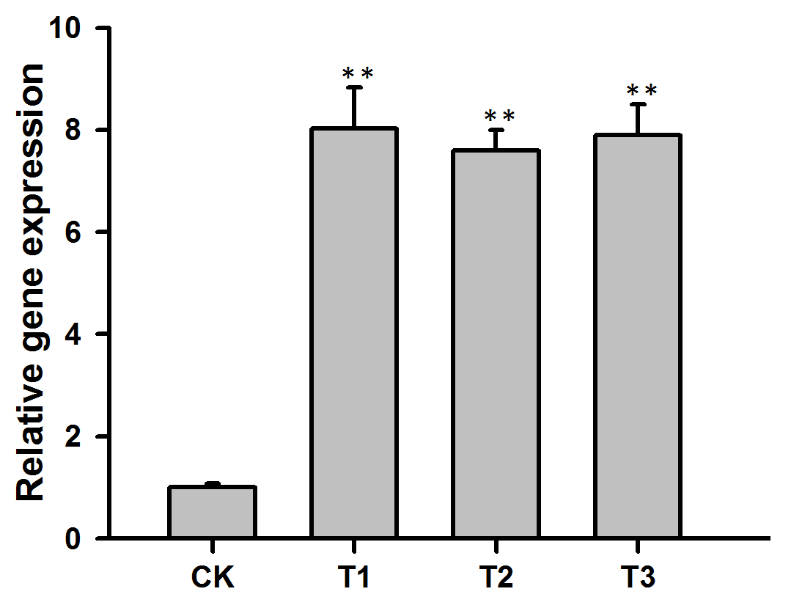


Fig. S5 Analysis of gene expression of *Ma1* in apple calli using qRT-PCR. CK indicates wild type, while T1, T2 and T3 represent transgenic lines. Asterisks indicate significant differences between transgenic lines and wild-type (t-test, *P* < 0.01).
